# Modulation of Ferroelectric and Optical Properties of La/Co-Doped KNbO_3_ Ceramics

**DOI:** 10.3390/nano11092273

**Published:** 2021-09-01

**Authors:** Xue Zhang, Ruijuan Qi, Shangwei Dong, Shuai Yang, Chengbin Jing, Lin Sun, Ye Chen, Xuekun Hong, Pingxiong Yang, Fangyu Yue, Junhao Chu

**Affiliations:** 1 Key Laboratory of Polar Materials and Devices (MOE), Department of Electronic Sciences, East China Normal University, Shanghai 200241, China; 52191213020@stu.ecnu.edu.cn (X.Z.); rjqi@ee.ecnu.edu.cn (R.Q.); 51191213049@stu.ecnu.edu.cn (S.D.); 52204700042@stu.ecnu.edu.cn (S.Y.); cbjing@ee.ecnu.edu.cn (C.J.); lsun@ee.ecnu.edu.cn (L.S.); ychen@ee.ecnu.edu.cn (Y.C.); pxyang@ee.ecnu.edu.cn (P.Y.); jhchu@clpm.ecnu.edu.cn (J.C.); 2 School of Electronic and Information Engineering, Changshu Institute of Technology, Changshu 215500, China; 3 National Laboratory of Infrared Physics, Shanghai Institute of Technical Physics, Shanghai 200083, China

**Keywords:** lead-free ferroelectrics, ceramics, microstructure, optical properties

## Abstract

The phase transition, microscopic morphology and optical and ferroelectric properties are studied in a series of La- and Co-doped KNbO_3_-based ceramics. The results show that the doping induces the transformation from the orthorhombic to the cubic phase of KNbO_3_, significantly reduces the optical bandgap and simultaneously evidently improves the leakage, with a slight weakening of ferroelectric polarization. Further analysis reveals that (i) the Co doping is responsible for the obvious reduction of the bandgap, whereas it is reversed for the La doping; (ii) the slight deterioration of ferroelectricity is due to the doping-induced remarkable extrinsic defect levels and intrinsic oxygen vacancies; and (iii) the La doping can optimize the defect levels and inhibit the leakage. This investigation should both provide novel insight for exploring the bandgap engineering and ferroelectric properties of KNbO_3_, and suggest its potential applications, e.g., photovoltaic and multifunctional materials.

## 1. Introduction

In the past several decades, ferroelectric materials have become important candidates for photovoltaic, random-access memory and optoelectronic devices due to their inherent spontaneous polarization [1,2,3,4,5,6]. Among them, KNbO_3_ (KNO) materials with excellent properties are a research hotspot. KNO is non-toxic and has become a potential photovoltaic material due to its great photoelectric properties, nonlinear optical coefficient, light refraction and other optical properties [7]. However, KNO, like most ferroelectric materials, has a wide optical bandgap (*E_g_* > 3 eV) and a low utilization rate of sunlight, and therefore the problem of how to increase its absorption response to visible light has become a popular research direction for KNO materials [8], e.g., by doping or ion substitution or composition, which are effective methods to adjust the optical bandgap and electrical property, as representatively reported with Mn doping by Manikandan et al. [9] and with Cr doping by Raja et al. [10]. Most seriously, the large leakage current of pure KNO is a key issue, which should intrinsically hinder its application as perovskite ferroelectric materials [11,12].

Here, K_1−*x*_La*_x_*Nb_1−*x*_Co*_x_*O_3_ (*x*KLNCO) ceramics with 0 ≤ *x* ≤ 0.12 are synthesized by doping KNO with a La- and Co-doping-dependent structure, while the morphology, optical *E_g_* and ferroelectricity have been investigated using X-ray diffraction (XRD), Raman spectroscopy, scanning electronic microscopy (SEM) imaging, absorption and photoluminescence (PL) spectroscopy and a ferroelectric hysteresis loop. The results reveal that the La doping sacrifices part of the ferroelectricity in order to optimize its leakage performance and improves the overall ferroelectric properties, although it inhibits the growth of the crystal. Co doping can significantly reduce the *E_g_* and enhance the optical absorption response to visible light due to the generation of new electronic level states within the gap of KNO. This emphasizes a great impact of doping on the energetic structure engineering and ferroelectricity of KNO-based ceramics, and thus provides new options for its multi-functional applications in optoelectronics and ferroelectrics.

## 2. Materials and Methods

The raw materials of K_2_CO_3_ (AR), Nb_2_O_5_ (AR), La_2_O_3_ (AR) and CoO (AR) are synthesized into a new type of perovskite ceramic *x*KLNCO (0 ≤ *x* ≤ 0.12 with an increase step of 0.03) through the solid-phase reaction method. Among them, the concentrations of K and Nb are both (1 − *x*) percent, and those of La and Co are both *x* percent. In order to compensate for the volatilization of potassium during high temperature sintering, 5% excess of K_2_CO_3_ was added to each composition. These raw materials were ball milled for 12 h to thoroughly mixed, and then pre-sintered at 700 °C for 6 h to form the precursor powder. Then they were ball milled again, and the powder was compressed into pellets with 5wt% polyvinyl alcohol binder. The pellets were first heated in air at 600 °C for 2 h to burn out the binder, and finally sintered at the optimum temperature of 1000 °C for 4 h. The relative density of the pure sample (KNO) was approximately 53.823%.

The XRD (Bruker D8 Advance, Karlsruhe, Germany) patterns and Raman (Horiba LabRam 800, Kyoto, Japan) spectra were used to investigate the crystalline structure characteristics of the prepared ceramics. The surface morphologies were observed by SEM (Zeiss Gemini 450, Oberkochen, Germany). The optical absorption was measured with an ultraviolet-visible-near infrared spectrophotometer (Varian Cary500, Palo Alto, CA, USA) equipped with an integrating sphere. The photoluminescence spectra were recorded on a Bruker Vertex 80v (Karlsruhe, Germany) for the near infrared waveband and PerkinElmer LS55 (Waltham, MA, USA) for the UV–Vis waveband, with the excitation wavelengths of 532 nm and 325 nm, respectively. The hysteresis loops were measured by a ferroelectric tester (Radiant Precision Premier II, Albuquerque, NM, USA) at an alternating frequency *f* = 1 kHz.

## 3. Results and Discussion

### 3.1. Structure

**Figure 1:**
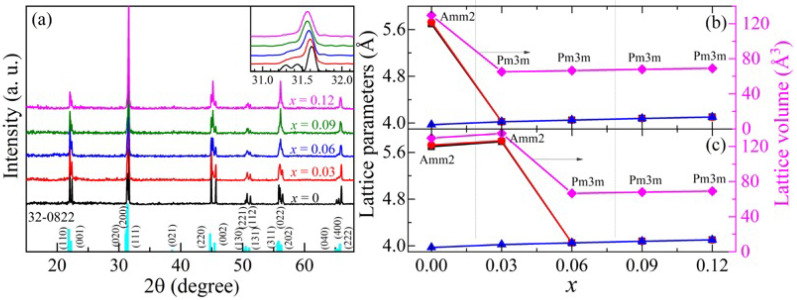
(**a**) XRD patterns of *x*KLNCO ceramics. The inset shows magnified patterns around 2θ~31.5°. The cyan lines at the bottom are indexed from JCPDS No. 32-0822, space group Amm2. (**b**) Lattice parameters (a—black, b—red, c—blue) and unit cell volume (V—pink) of *x*KLNCO (with Amm2 phase for *x* = 0 and Pm3m phase for *x* = 0.03~0.12). (**c**) The calculated values of *x* = 0.03 are obtained by considering the mixed phase as the Amm2 phase to make a comparison. Assuming V = a × b × c.

Figure 1a shows the room temperature XRD patterns for *x*KLNCO (0 ≤ *x* ≤ 0.12) ceramics. The XRD pattern of the sample with *x* = 0 is basically consistent with the standard ICDD card No.32-0822, indicating that the Bragg reflections for the KNO ceramic are indexed to the orthorhombic crystal system of the space group *Amm2* [13]. The inset in the figure enlarges the part of 2*θ* ≈ 31.5°, and it is observed that the three diffraction peaks of the pure KNO gradually merge into one diffraction peak with the increase in *x*, which results in the appearance of the cubic phase [14]. Notice that at *x* = 0.03, 0.06 and 0.09, the diffraction peaks (200) and (220) exhibit significant asymmetry and have wider shoulders at the low-angle side. This is evidence of the existence of a weak tetragonal ferroelectric phase [15]. The single peaks of XRD diffraction of the doped samples are asymmetric, which reveals a clear lattice distortion away from the perfect cubic symmetry. These suggest that *x*KLNCO crystals have undergone a series of structural phase transitions, i.e., from the orthorhombic to tetragonal phase, and then to the cubic phase. It can be clearly observed that as the doping level (*x* value) increases, the main diffraction peaks shift toward lower 2*θ* angles. This is most likely because the doped Co^2+^ (0.745 Å) has a larger ionic radius than Nb^5+^ (0.64 Å). Doping with larger radius ions will cause the lattice to expand; according to the Bragg diffraction formula *2dsinθ = nλ*, the diffraction angle *θ* will decrease [16]. Figure 1b illustrates the variations of lattice parameters and unit cell volumes with *x*-value. Lattice parameters for KNO are calculated to be a = 5.69589 Å, b = 5.72433 Å and c = 3.97285 Å. They (a, b and c) gradually evolve to be equal as the *x*-value increases, which is consistent with the transition from the orthorhombic to cubic phase. According to the Scherrer formula, the average size of the crystal grains is calculated to be 58 nm.

**Figure 2:**
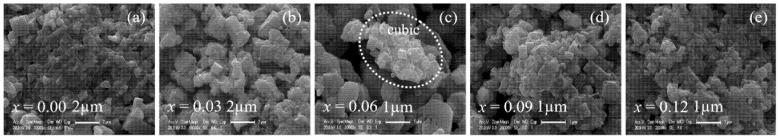
SEM images of *x*KLNCO ceramics with different compositions: (**a**) *x* = 0, (**b**) *x* = 0.03, (**c**) *x* = 0.06, (**d**) *x* = 0.09, (**e**) *x* = 0.12.

Figure 2a–e show the microstructures of as-prepared *x*KLNCO by SEM. From the surface morphologies, the ceramics are well-crystallized, since favorable particles can be identified clearly. However, the voids on the surface of the *x*KLNCO appear, of which the reason may be attributed to the K volatilization. As the doping increases, the surface morphologies of the *x*KLNCO exhibit an evident decrease in the average grain size, indicative of the doping-composition-dependence of the grain growth. At the same time, the laminar structure can be observed in the samples of *x* ≥ 0.03, as clearly shown in Figure 2b,c. When *x* = 0.06, besides the obvious cubic appearances, there are also some imperfect symmetric crystals, which suggests a state of a mixed structure of crystals at this condition.

**Figure 3:**
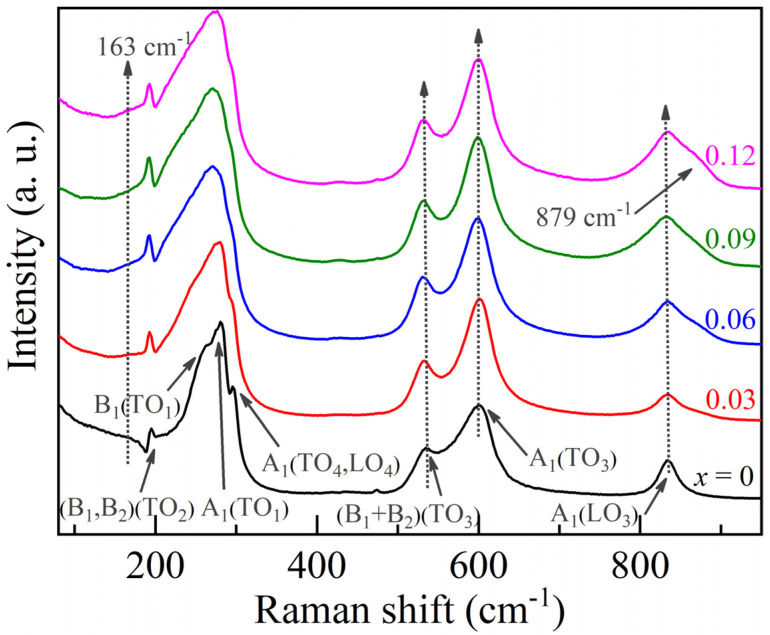
Raman scattering spectra of *x*KLNCO ceramics at room temperature.

Raman spectra are further measured for confirming the local lattice distortion by analyzing the structural and lattice vibration. From the group theory analysis [17], it exhibits 12 optical modes of 4A_1_ + 4B_1_ + 3B_2_ + A_2_ symmetries for space group $C_{2v}^{14}$ (*Amm*2). In these 12 models, A_2_ is Raman-active, and the rest of the models are both Raman- and infrared-active. Figure 3 shows the room-temperature Raman spectra of *x*KLNCO (0.0 ≤ *x* ≤ 0.12) ceramics, where nine characteristic vibration modes appear. Among them, TO_1_, TO_3_, TO_4_, LO_3_ and LO_4_ are the transverse/longitudinal optical (TO/LO) phonon modes, reflecting the NbO_6_ octahedral polarization lattice vibration. The A_1_(TO_1_) mode is at ∼281 cm^−1^, of which the shoulders on both sides are the B_1_(TO_1_) and A_1_(TO_4_, LO_4_) modes. The A_1_(TO_3_) mode appears at ∼602 cm^−1^, the A_1_(LO_3_) mode is found at ∼834 cm^−1^ with a low intensity and the (B_1_ + B_2_)(TO_3_) mixed mode at ∼535 cm^−1^ is associated with the vibration of the octahedral [18]. Two modes (B_1_, B_2_)(TO_2_) degenerated at ∼195 cm^−1^ are associated with the vibration of the Nb-O bonds in the octahedral. In the Raman spectrum of KNO, the low-wavenumber region below ~500 cm^−1^ is mainly related to the BO_6_ bending vibration mode A_1_(TO_1_) and the two spike modes TO_2_ and TO_4_, which confirm the orderly existence of long-range polarization [19]. Beyond ~500 cm^−1^, the vibration mode at ~834 cm^−1^ is related to the perovskite structure of KNO [20]. For the Raman spectrum of *x*KLNCO, it is observed that the vibration mode in the high-wavenumber range (>500 cm^−1^) red-shifts, and the relative intensity of the vibration peak increases. With the increase in the doping level, a vibration at ~163 cm^−1^ appears, which is related to the vibrational change in the Nb-O bond in the oxygen octahedron. This indicates that excess cations appear in the nano-regional lattice, replacing K^+^ with La^3+^, which is equivalent to adding two +1 valent ions at the A site [14]. In addition, a weaker vibration appeared at ~879 cm^−1^, the A_1_(TO_1_) mode gradually expanded and the two modes at 263 cm^−1^ and 296 cm^−1^ gradually disappeared. The relative intensity of the vibration modes at 535 cm^−1^ and 602 cm^−1^ also changed, and the (B_1_ + B_2_)(TO_3_) mode gradually increased. These modes are all related to the expansion and flexural vibration of the NbO_6_ octahedron. Theoretically, the vibration of the NbO_6_ octahedron consists of 1A_1g_ (*υ*_1_) + 1E_g_ (*υ*_2_) + 2F_1u_ (*υ*_3_, *υ*_4_) + F_2g_ (*υ*_5_) + F_2u_ (*υ*_6_) modes. Among them, 1A_1g_ (*υ*_1_) + 1E_g_ (*υ*_2_) + 2F_1u_ (*υ*_3_) is the stretching vibration mode, and the rest are bending modes [21]. Therefore, based on the changes in the *υ*_1_, *υ*_2_ and *υ*_5_ modes, it is inferred that the increase in the doping level will reduce the degree of distortion along the polar axis; that is, the ferroelectric polarization strength of *x*KLNCO ceramics will be weakened.

### 3.2. Optical and Ferroelectric Properties

**Figure 4:**
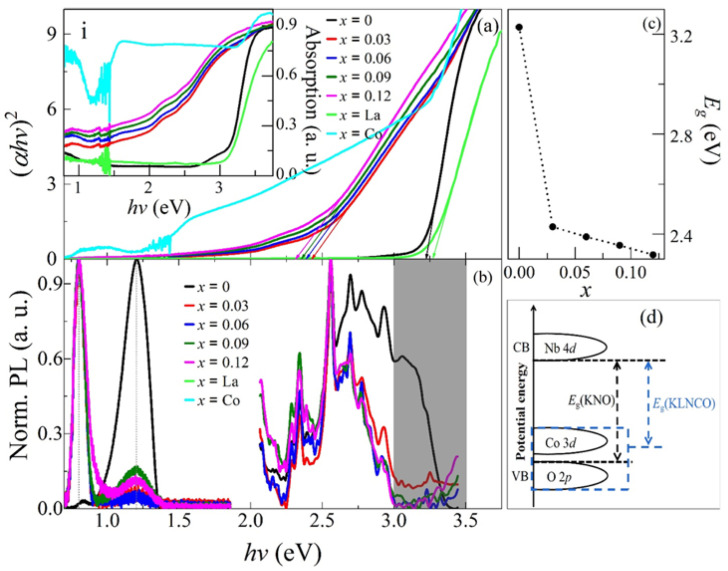
(**a**) Plots of (α*hv*)^2^ versus *hv* for the absorption. Insets (i) shows absorption spectra of *x*KLNCO ceramics; (**b**) normalized PL spectra excited at the 325 nm and 405 nm, respectively; (**c**) the change in band gap with doping amount *x*; (**d**) the schematic diagram of the energy band structure change principle of *x*KLNCO ceramics.

Figure 4 gives the optical results of *x*KLNCO, including absorption (a) and PL (b) in a wide wavelength range of 0.7–3.75 eV. The inset (i) in Figure 4a demonstrates that the absorption edges of all *x*KLNCO samples present a red-shift with the increase in the *x*-value. The *E_g_* with different *x*-values can be estimated from the Tauc plot of (*αhν*)^2^ vs. *hν* for the direct *E_g_* material in Figure 4a, where *α* is the absorption coefficiency and *hν* is the photon energy [3]. As shown in Figure 4c, the *E_g_* is ~3.22 eV, 2.42 eV, 2.38 eV, 2.35 eV and 2.31 eV, for *x* = 0, 0.03, 0.06, 0.09 and 0.12, respectively. It should be pointed out that for the sample with *x* = 0 (KNO), the absorption only occurs when the photon energy exceeds ~2.75 eV, while for the doped KNO (*x*KLNCO), the start energy point is largely red-shifted and almost extended to the low energy position at ~1.5 eV; see the inset (i) in Figure 4a, which, together with the narrowed-*E*_g_, could be a benefit for its application in perovskite oxide-based photovoltaics. These results suggest that La and Co doping can enhance the ability of KNO to absorb visible photons. In order to further study the reasons for the *E_g_* narrowing, two samples with *x* = 0.09 by either La or Co doping are prepared. It is found that doping with Co not only induces a significant reduction of *E_g_*, but also introduces shallow impurity levels. This is due to the incorporation of Co^2+^ ions to form a new electronic state, which changes the top of the valence band from O 2*p* in KNO to a hybrid energy level formed by the hybridization of Co 3*d* and O 2*p* orbitals [22]. Figure 4d schematically shows the band structure of KNO and *x*KLNCO crystals. However, the incorporation of La widens the *E_g_* and the introduction of Co optimizes the shallow defects. This phenomenon can be explained from the Burstein–Moss (BM) effect [23]. In other words, in doped samples, the donor electrons occupy the bottom states of the conduction band, and the incorporation of La^3+^ causes the Fermi level of KNO to enter into the conduction band and the donor electrons to fill the bottom of the conduction band. According to Pauli’s exclusion principle, the transition of electrons from the top of the valence band to the bottom of the conduction band will need to absorb more energy, which proves the broadening of the optical *E_g_*. More evidence can be seen in PL spectra shown in Figure 4b. The broad emission band with multiple sharp peaks in the right side in Figure 4b is intrinsically from different electronic states of KNO (*x* = 0). It is observed that an absorption in the range of 2.75~3.45 eV shows an evident dependence of doping, i.e., becoming weak and finally disappearing with the increase in thedoping level. It is proximate to the *E_g_*-transition, which can be attributed to the high band-tail states induced by the Co doping, and the orbital hybridization occurs. Energetically, the PL part of 2~2.75 eV with separately sharp peaks corresponds to shallow defect-related signals, but the doping does not almost affect the emission position and intensity. It may be an intrinsic luminescence of the (NbO_6_)^−^ group that leads to the electronic charge in the octahedral position of KNO to migrate from oxygen ions to niobium ions during the excitation process, thereby deforming the NbO_6_ octahedron and additionally affecting the polarization of KNO, as discussed by Raja et al. [10]. As for the emission around 1.2 eV at the left side in Figure 4b, it is already a very deep impurity level. It is interesting to point out that the doping can make the emission of this deep level disappear, but can also enhance the signal of a deeper defect at ~0.8 eV (which has already been observed in the pure KNO), of which the mechanism needs further investigation.

**Figure 5:**
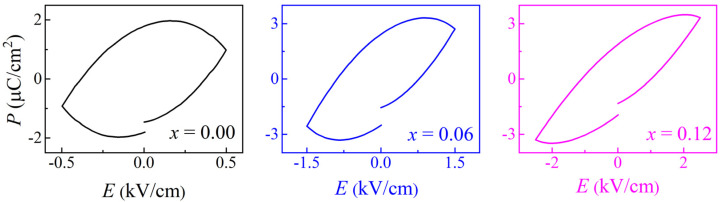
*P*–*E* hysteresis curves of *x*KLNCO ceramics.

Figure 5 shows the *P-E* curves of all samples in order to reveal the effect of doping on the ferroelectric properties. It shows that the leakage gradually improves as the doping increases. For perovskite oxide potassium niobate, the ferroelectricity originates from the displacement of Nb^5+^ deviating from the center of the symmetry of the NbO_6_ octahedron, which leads to the destruction of the spatial inversion symmetry. Macroscopically, it manifests the inconsistent positive and negative charge centers caused by lattice distortion [24]. As the samples transfer to the cubic phase structure after doping, the symmetry is enhanced and the polarization of the carriers is weakened. That is, the deterioration of ferroelectricity also indicates that the samples undergo a phase transition from orthogonal to cubic as the *x*-value increases. When the Co^2+^ replaces part of Nb^5+^ in the KNO lattice, it will cause octahedral deformation. From the Raman results, this deformation will reduce the degree of distortion along the polar axis. Therefore, the ferroelectric polarization is weakened after doping. The electric hysteresis loop of pure KNO is relatively round and the left and right sides appear asymmetrical, which is caused by the large leakage generated by the inherent oxygen defects in KNO ceramics. The leakage phenomenon is improved after doping, which may be due to a rare earth modification. After doping, the average size of the crystal decreases, and the local interface energy increases. Under the role of the electric field, the energy barrier is reduced, which simplifies the ferroelectric domain switching [25]. Therefore, it is speculated that La doping will produce a soft ferroelectric effect, which will slightly improve the performance [25]. The reduction of the leakage may also be a reflection of the improvement of KNO’s intrinsic defects, which is consistent with the results of the PL spectroscopy.

## 4. Conclusions

In summary, the dependence of microscopic morphology, lattice vibration, optical bandgap, defects and ferroelectric properties in *x*KLNCO with the doping level has been studied in details. With the increase in *x*-value from 0 to 0.12, the grain size decreases significantly, and the optical *E_g_* drops from 3.22 to 2.31 eV. Simultaneously, the ferroelectric polarization is weakened but the leakage phenomenon is improved. These characteristics greatly enhance the response of *x*KLNCO ceramics to visible light, providing a potential application in photovoltaics, and can also meet the needs of solid-state devices, such as random-access memory.

## Data Availability

The data presented in this study are available on request from the corresponding author.
