# Correction: Zhang et al. Modulation of Ferroelectric and Optical Properties of La/Co-Doped KNbO_3_ Ceramics. *Nanomaterials* 2021, *11*, 2273

**DOI:** 10.3390/nano16020109

**Published:** 2026-01-14

**Authors:** Xue Zhang, Ruijuan Qi, Shangwei Dong, Shuai Yang, Chengbin Jing, Lin Sun, Ye Chen, Xuekun Hong, Pingxiong Yang, Fangyu Yue, Junhao Chu

**Affiliations:** 1Key Laboratory of Polar Materials and Devices (MOE), Department of Electronic Sciences, East China Normal University, Shanghai 200241, China; 52191213020@stu.ecnu.edu.cn (X.Z.); rjqi@ee.ecnu.edu.cn (R.Q.); 51191213049@stu.ecnu.edu.cn (S.D.); 52204700042@stu.ecnu.edu.cn (S.Y.); cbjing@ee.ecnu.edu.cn (C.J.); lsun@ee.ecnu.edu.cn (L.S.); ychen@ee.ecnu.edu.cn (Y.C.); pxyang@ee.ecnu.edu.cn (P.Y.); jhchu@clpm.ecnu.edu.cn (J.C.); 2School of Electronic and Information Engineering, Changshu Institute of Technology, Changshu 215500, China; 3National Laboratory of Infrared Physics, Shanghai Institute of Technical Physics, Shanghai 200083, China

## Error in Figure

In the original publication [1], there was an error in Figure 1 as it was published. The lattice cell volume in Figure 1b was marked incorrectly, and the lattice parameter and volume of *x* = 0.03 with different phases (Amm2 or Pm3m) to make a comparison were missing. The corrected Figure 1 appears below.

**Figure 1 nanomaterials-16-00109-f001:**
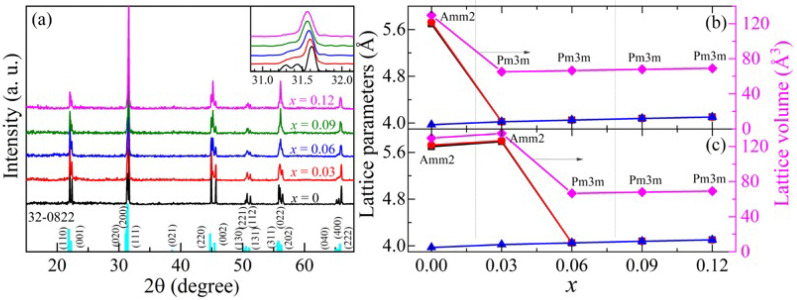
(**a**) XRD patterns of *x*KLNCO ceramics. The inset shows magnified patterns around 2θ~31.5°. The cyan lines at the bottom are indexed from JCPDS No. 32-0822, space group Amm2. (**b**) Lattice parameters (a—black, b—red, c—blue) and unit cell volume (V—pink) of *x*KLNCO (with Amm2 phase for *x* = 0 and Pm3m phase for *x* = 0.03~0.12). (**c**) The calculated values of *x* = 0.03 are obtained by considering the mixed phase as the Amm2 phase to make a comparison. Assuming V = a × b × c.

In the original publication [1], there was an error in Figure 4 as it was published. The curve color of *x* = 0.09 and *x* = 0.12 in the inset in Figure 4a was reversed. The corrected Figure 4 appears below.

**Figure 4 nanomaterials-16-00109-f004:**
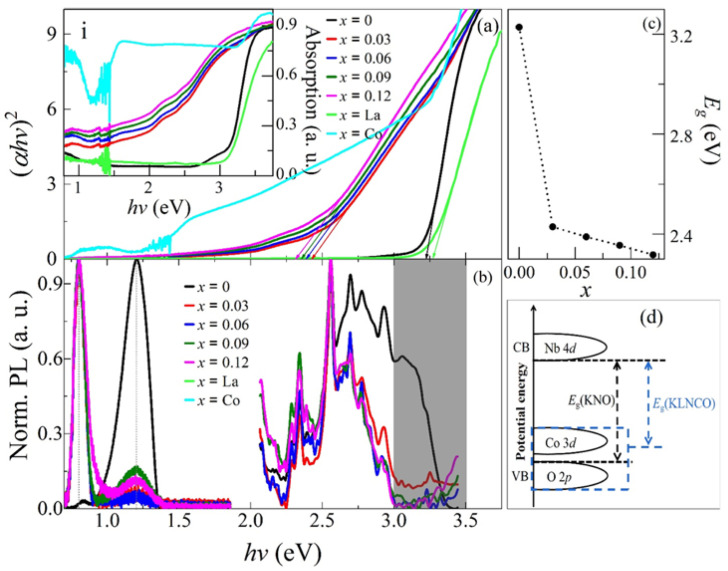
(**a**) Plots of (α*hv*)^2^ versus *hv* for the absorption. Insets (i) shows absorption spectra of *x*KLNCO ceramics; (**b**) normalized PL spectra excited at the 325 nm and 405 nm, respectively; (**c**) the change in band gap with doping amount *x*; (**d**) the schematic diagram of the energy band structure change principle of *x*KLNCO ceramics.

## Text Correction

There was an error in the original publication [1], which involved a lack of instrument details provided. A correction has been made to 2. Materials and Methods, Paragraph 2, and should read:

The XRD (Bruker D8 Advance, Karlsruhe, Germany) patterns and Raman (Horiba LabRam 800, Kyoto, Japan) spectra were used to investigate the crystalline structure characteristics of the prepared ceramics. The surface morphologies were observed by SEM (Zeiss Gemini 450, Oberkochen, Germany). The optical absorption was measured with an ultraviolet-visible-near infrared spectrophotometer (Varian Cary500, Palo Alto, CA, USA) equipped with an integrating sphere. The photoluminescence spectra were recorded on a Bruker Vertex 80v (Karlsruhe, Germany) for the near infrared waveband and PerkinElmer LS55 (Waltham, MA, USA) for the UV–Vis waveband, with the excitation wavelengths of 532 nm and 325 nm, respectively. The hysteresis loops were measured by a ferroelectric tester (Radiant Precision Premier II, Albuquerque, NM, USA) at an alternating frequency *f* = 1 kHz.

## Missing Citation

In the original publication, Reference [21] “MOSS, T.S. Theory of the Spectral Distribution of Recombination Radiation from InSb. *Proc. Phys. Soc. Sect. B* **1957**, *70*, 247–250” was wrongly cited. This reference has been replaced with reference [14] “Zhou, W.; Deng, H.; Yang, P.; Chu, J. Structural phase transition, narrow band gap, and room-temperature ferromagnetism in [KNbO_3_]_1−x_[BaNi_1/2_Nb_1/2_O_3−δ_]_x_ ferroelectrics. *Appl. Phys. Lett.* **2014**, *105*, 111904” in 3. Results and Discussion, 3.1. Structure, Paragraph 3, and should read:

Raman spectra are further measured for confirming the local lattice distortion by analyzing the structural and lattice vibration. From the group theory analysis [17], it exhibits 12 optical modes of 4A_1_ + 4B_1_ + 3B_2_ + A_2_ symmetries for space group $C_{2v}^{14}$ (*Amm*2). In these 12 models, A_2_ is Raman-active, and the rest of the models are both Raman- and infrared-active. Figure 3 shows the room-temperature Raman spectra of *x*KLNCO (0.0 ≤ *x* ≤ 0.12) ceramics, where nine characteristic vibration modes appear. Among them, TO_1_, TO_3_, TO_4_, LO_3_ and LO_4_ are the transverse/longitudinal optical (TO/LO) phonon modes, reflecting the NbO_6_ octahedral polarization lattice vibration. The A_1_(TO_1_) mode is at ∼281 cm^−1^, of which the shoulders on both sides are the B_1_(TO_1_) and A_1_(TO_4_, LO_4_) modes. The A_1_(TO_3_) mode appears at ∼602 cm^−1^, the A_1_(LO_3_) mode is found at ∼834 cm^−1^ with a low intensity and the (B_1_ + B_2_)(TO_3_) mixed mode at ∼535 cm^−1^ is associated with the vibration of the octahedral [18]. Two modes (B_1_, B_2_)(TO_2_) degenerated at ∼195 cm^−1^ are associated with the vibration of the Nb-O bonds in the octahedral. In the Raman spectrum of KNO, the low-wavenumber region below ~500 cm^−1^ is mainly related to the BO_6_ bending vibration mode A_1_(TO_1_) and the two spike modes TO_2_ and TO_4_, which confirm the orderly existence of long-range polarization [19]. Beyond ~500 cm^−1^, the vibration mode at ~834 cm^−1^ is related to the perovskite structure of KNO [20]. For the Raman spectrum of *x*KLNCO, it is observed that the vibration mode in the high-wavenumber range (>500 cm^−1^) red-shifts, and the relative intensity of the vibration peak increases. With the increase in the doping level, a vibration at ~163 cm^−1^ appears, which is related to the vibrational change in the Nb-O bond in the oxygen octahedron. This indicates that excess cations appear in the nano-regional lattice, replacing K^+^ with La^3+^, which is equivalent to adding two +1 valent ions at the A site [14]. In addition, a weaker vibration appeared at ~879 cm^−1^, the A_1_(TO_1_) mode gradually expanded and the two modes at 263 cm^−1^ and 296 cm^−1^ gradually disappeared. The relative intensity of the vibration modes at 535 cm^−1^ and 602 cm^−1^ also changed, and the (B_1_ + B_2_)(TO_3_) mode gradually increased. These modes are all related to the expansion and flexural vibration of the NbO_6_ octahedron. Theoretically, the vibration of the NbO_6_ octahedron consists of 1A_1g_ (*υ*_1_) + 1E_g_ (*υ*_2_) + 2F_1u_ (*υ*_3_, *υ*_4_) + F_2g_ (*υ*_5_) + F_2u_ (*υ*_6_) modes. Among them, 1A_1g_ (*υ*_1_) + 1E_g_ (*υ*_2_) + 2F_1u_ (*υ*_3_) is the stretching vibration mode, and the rest are bending modes [21]. Therefore, based on the changes in the *υ*_1_, *υ*_2_, and *υ*_5_ modes, it is inferred that the increase in the doping level will reduce the degree of distortion along the polar axis; that is, the ferroelectric polarization strength of *x*KLNCO ceramics will be weakened.

## References

With this correction, the order of some references has been adjusted accordingly. The authors state that the scientific conclusions are unaffected. This correction was approved by the Academic Editor. The original publication has also been updated.
